# A pressure monitoring approach for pressure ulcer prevention

**DOI:** 10.1186/s42490-023-00074-6

**Published:** 2023-08-11

**Authors:** Bethel Osuagwu, Euan McCaughey, Mariel Purcell

**Affiliations:** 1 Biomedical Engineering Research Division, School of Engineering, University of Glasgow, Glasgow, UK; 2 Scottish Centre for Innovation in Spinal Cord Injury, Queen Elizabeth National Spinal Injuries Unit, Queen Elizabeth University Hospital, Glasgow, UK; 3 Queen Elizabeth National Spinal Injuries Unit, Queen Elizabeth University Hospital, Glasgow, G51 4TF Scotland; 4 Neuroscience Research Australia, Sydney, Australia

**Keywords:** Pressure ulcer, Monitoring, Prevention

## Abstract

**Background:**

A pressure ulcer (PU) is a debilitating condition that disproportionately affects people with impaired mobility. PUs facilitate tissue damage due to prolonged unrelieved pressure, degrading quality of life with a considerable socio-economic impact. While rapid treatment is crucial, an effective  prevention strategy may help avoid the development of PUs altogether. While pressure monitoring is currently used in PU prevention, available monitoring approaches are not formalised and do not appropriately account for accumulation and relief of the effect of an applied pressure over a prolonged duration. The aim of this study was to define an approach that incorporates the accumulation and relief of an applied load to enable continuous pressure monitoring.

**Results:**

A tunable continuous pressure magnitude and duration monitoring approach that can account for accumulated damaging effect of an applied pressure and pressure relief over a prolonged period is proposed. Unlike classic pressure monitoring approaches, the presented method provides ongoing indication of the net impact of a load during and after loading.

**Conclusions:**

The tunable continuous pressure magnitude and duration monitoring approach proposed here may further development towards formalised pressure monitoring approaches that aim to provide information on the risk of PU formation in real-time.

## Background

Pressure ulcer (PU) is a localised damage to the skin and/or underlying tissues resulting from excessive pressure, friction and shearing forces particularly over bony prominences [1, 2]. PUs may begin from the skin and progress into deep tissues or, in the serious case known as deep tissue injury (DTI), damage the deep tissues first before presenting any visually detectable sign on the skin [3]. PUs have a detrimental effect on quality of life and a significant socio-economic impact [1]. They complicate a person’s rehabilitation programme and are a deterrent to participation in social and activities of daily living [1].

Several risk factors are associated with PU development, including spinal cord injury (SCI), moisture, poor nutrition and immobility. Particularly, people with SCI have increased risk such that almost all are expected to develop a serious case over their lifetime [1]. The risk is often predicted using a scale such as the Braden scale which encompasses sensory perception, mobility, nutrition, moisture, friction & shear, and activity [4].

Various mechanisms contribute to PU formation. The main initiator is sustained mechanical loading which leads to compressive tissue straining. There are several theories on the plausible mechanisms through which the sustained mechanical loading results in PU. First, friction or shear forces at the skin-support surface interface may lead to a direct skin damage. The direct damage may weaken the skin which exposes the underlying deeper tissues such that they become more susceptible to damage through pressure or infection. This allows the damage to propagate from the skin into the deeper tissues. Second, substantial mechanical loading of sufficient magnitude causes a localised occlusion of blood vessels leading to deprivation of supplies to a tissue. This localised ischemia is often considered as one of the most important factors in PU formation as the deprivation of supplies for a prolonged period (in hours [5]) leads to cell death [6–8]. The reperfusion of the tissue post-ischaemia mediates ischaemia reperfusion injury which builds on or even accelerates the damage on the post-ischemic tissue [8, 9]. Third, just as mechanical loading leads to occlusion of the blood vessels, it leads to the occlusion of the lymphatic system resulting in cellular damage due to accumulation of cellular waste products in the interstitial fluid [10]. Finally, sustained mechanical loading leads to sustained cell deformation and direct damage [8, 11, 12] in addition to the damage relating to friction and shear forces. While ischaemia related injury occurs at a time scale of hours [5], a direct cell deformation can occur within minutes [5, 13]. This type of damage may primarily cause DTIs near bony prominences which have largest internal stress as a result of mechanical loading.

The socio-economic impact means that elaborate clinical guidelines and significant research effort are dedicated to preventing PU. Prevention strategies deserve attention since current PU early detection methods such as subepidermal moisture scanning technology and ultrasonography rely on the development of injury [14] to a certain degree which is required to detect signs. Development of a formalised robust monitoring system that can predict and prevent PU development altogether seems essential. Such a monitoring system may also indicate when an early detection assessment is required and can even desirably reduce the need for the assessment. One method of PU prevention is *pressure monitoring* where signals such as interface pressure and/or internal stress measurement due to a mechanical load is used for evaluation and recommendation of support surfaces, pressure relief manoeuvres and general repositioning. Since pressure significantly facilitates PU development through multiple mechanisms as previously highlighted, pressure monitoring has received much interest as a preventative method as early as the 1970s [15]. Adequate pressure monitoring to indicate the rate, duration and type of pressure redistribution and relief will not only reduce the incidence of PU but will also make the management of the condition efficient. Current monitoring strategies primarily aim to identify body regions experiencing high pressure magnitude e.g. above 32 mmHg [16–19] and provide this information for evaluation and recommendation purposes. Less attention is given to the duration of application of the pressure despite its relevance as regards to PU development.

Gefen argued that avoiding high pressure magnitude such as the 32 mmHg or greater interface pressure may not account for pressure relief due to higher internal stress and/or misuse of capillary pressure [16]. It should be particularly noted that as well as interface pressure magnitude and effective internal stress, the duration of application is an important factor in PU development [8, 20–22]. According to pressure–time-injury threshold curve an inverse [23] or more recently a sigmoid [24, 25] relationship is said to exists between injury-causing pressure magnitude and duration of application; where a small magnitude pressure may require a long time to cause tissue damage. Accordingly, for a large pressure magnitude, only a short application period is required. Therefore it is important to consider the pressure magnitude as well as the application duration together for an effective pressure monitoring.

Furthermore pressure monitoring should be performed continuously to indicate in real-time the risk of PU development on a tissue considering its health status based on the net effect of applied load (where the net effect is the accumulated effect over time, minus relief over the same period). The current technique, in pressure magnitude and duration monitoring, for representing the risk of PU of a tissue rely on the integration of the effective pressure/stress with respect to time [22, 26]. Although this quantifies the pressure/stress dose and is a useful indicator of the risk of PU development for a loaded tissue, it does not solely quantify/represent tissue damage and importantly the application method do not properly take pressure relief over time into account.

There is currently no formalised approach for pressure magnitude and duration monitoring with respect to accumulation and relief of the impact of applied pressure continuously over a prolonged period. This meant that authors have used various approaches to implement pressure monitoring (see [27] for different implementation in different devices). For example some authors have used the moving average method while others have used integration to accumulate a representative effect, e.g. stress dose, of applied pressure on a tissue. In the case of relieving the ‘effect’ following pressure relief, moving average, fixed time and inverse time methods have been tried. The lack of standard makes the approaches difficult to analyse.

The objective of this study is to define an approach that incorporates both the accumulation and relief of the effect of an applied load to enable continuous pressure magnitude and duration monitoring over a prolonged period of time. Pulling together the remarkable findings and available data in PU research, the present work proposes a method of real-time processing of pressure/stress data with relation to data/model-based estimation of tissue damage and incorporation of pressure relief over time. Damaging effect and relief functions are defined to respectively represent an indication of tissue damage due to an applied load, and account for relief to continuously show the effective impact of the load. Also defined is a relief time used to tune the relief function. The presented method may provide the basis for further development to formalise pressure monitoring.

## Methods

### Proposed monitoring approach

Assuming that any given pressure (or internal strain or stress) over a duration has a *damaging effect* denoted by $I$ on a specified tissue with fixed characteristics. For simplicity, other factors that contribute to PU formation are also considered fixed. In discrete time the damaging effect $I(n)$ with the discrete time variable $n$, may be represented as,

1 $${I}_{g}\left(n\right)=g\left(t,P,I\left(n-1\right),n\right), n=1, 2, 3\dots$$

where the composed function $g(\cdot )$ is defined here as a *damaging effect function* that represents the ongoing damaging effect given pressure (or stress), $P$; pressure application duration, $t$ (which may be equivalent to the sampling period) and the previous damaging effect.

When the applied pressure is relieved, the current value of $I$ may decay according to an appropriately defined *relief function,* $d(\cdot)$ which determines the behaviour of the decay process. It may be assumed that $I$ is below a certain critical value above which recovery is impossible. Therefore, assuming that the fixed conditions remain, at any discrete time the proposed resultant abstract damaging effect is given by,

2 $$I\left(n\right)={I}_{g}(n)-d(\cdot )= g\left(t,P,I\left(n-1\right),n\right)-d\left(\cdot \right)$$

such that $d\equiv 0$ when $g$ is non-trivial. The possible functions for $g$ and $d$ are explored next.

#### Damaging effect function

An averaging filter [19] as well as an integrator [22, 26] have previously been used for $g$. An integrator is likely an appropriate choice for $g$ to accumulate the damaging effect over a prolonged period, thus,

3 $$g\left(t,P,I\left(n-1\right),n\right)= I\left(n-1\right)+q(t,P,n)$$

where the function $q$ explored in a later section, is a damaging effect estimator, in this case, a *pressure–time damaging effect estimator* for the present pressure magnitude $P$ applied over a duration $t$.

#### Relief function

Experimental data may be required to correctly model how a tissue would recover during pressure relief. Most models and data (e.g. see [25, 28] used in [29]) of PU relate to the formation of the damage rather than on recovery during pressure relief. In the absence of experimental data, a few functions have previously been explored in the literature [27]. Verbunt and Bartnect implicitly used an averaging method where the damaging effect would decay according to a moving averaging filter [19]. In Portnoy et al. 2011, [30], the damaging effect would decay proportionally to the time length (in seconds) of pressure relief. This can be achieved by multiplying an accumulated damaging effect by the inverse of the total relief period. Another approach is to set a fixed period after which all accumulated damaging effect decays to zero [15, 26]. Here, a continuous linear function and an exponential decay one are explored to implement more plausible relief functions.

##### Linear decay

With a linear function, decay occurs at a constant rate where $d$ can be chosen as,

4 $$d=\beta \left(1-U\right), U\ni 0\le U\le 1$$

The decay/relief rate $\beta$ (a coefficient of relief) relates to the rate of a tissue’s recovery following a pressure relief and $U$ is a parameter that determines the condition for relief such that its value is less than unity during pressure relief and it may be graded in proportion to the degree of the relief. For a maximum pressure relief, $U=0$.

##### Exponential decay

The approach is to relate the decay of the damaging effect following pressure relief to the previous damaging effect through a problem specific relief rate, $\beta$, such that $d$ may be chosen to get an exponential function,

5 $$d\left(I\left(n-1\right)\right)=\beta I\left(n-1\right)\left[1-U\right], U\ni 0\le U\le 1$$

The grading of the relief is achieved with the factor $[1-U]$ which scales $\beta$.

#### Summary

With $g$ as an integrator and considering the linear and exponential decay methods as options for $d$, the proposed monitoring approach given in Eq. 2 can be implemented as,

6 $$I\left(n\right)=\left\{\begin{array}{c}q\left(t,P,n\right)+I\left(n-1\right)-\beta \left(1-U\right) ,\\ q\left(t,P,n\right)+\left[1-\beta \left(1-U\right)\right]I\left(n-1\right) ,\end{array}\right. \begin{array}{c}if\;d\left(.\right)=\beta (1-U)\\ if\;d\left(.\right)=\beta \left(1-U\right)I(n-1)\end{array}$$

Equation 6 can be used continuously to simultaneously accumulate and relieve the total damaging effect for monitoring purposes. Crucially, the monitoring approach could be utilised to provide ongoing information on the impact of a load on a monitored tissue beyond loading rather than the classic current load on the tissue. If the total damaging effect surpasses a set limit at any given instance, an alarm may be raised.

### Smoothing factor and relief time

For the linear decay(i.e. the first case in Eq. 6), with an ideal pressure relief where $U=0$, from Eq. 6, we see that when $q(t,P,n)=0$ the accumulated damaging effect will decay linearly with a constant factor $\beta$,

7 $$I\left(n\right)=I\left(n-1\right)-\beta$$

The relief rate β, can be set by choosing a *relief time.* The relief time is related here to the time it takes for any accumulated damaging effect $I$ to decay to a harmless value. It has been used as far back as the ‘70s by Temes and Harder on a pressure relief training device [15]. More recent work by Linder-Ganz and colleagues assumed a relief time of 1 s in their detailed finite element analysis modelling study in humans [26]. When using internal compression stress, 2 kPa has been used as a harmless value for humans [26] guided by data from animal experiments [25]. The recommendation from Consortium for Spinal Cord Medicine (CSCM) is 2 min [1] based on tissue oxygen recovery time following pressure relief [31]. But this may need to be adjusted by considering the applied load and the current status of the tissue under monitor.

For the present linear decay case, $\beta =\frac{\Delta T}{{t}_{r}}$, where in this relation $\Delta T$ is a discrete sampling time interval (in seconds) and ${t}_{r}$ is the problem specific relief time. Example, for a relief time of 5 min, ${t}_{r}=300 s$. In this case, with a sampling time of 0.1 s, $\beta =0.00033$.

For the exponential decay(i.e. the second case in Eq. 6), with an ideal pressure relief where $U=0$, from Eq. 6, we see that when $q(t,P,n)=0$ the accumulated damaging effect will decay exponentially with a smoothing factor $\beta$,

8 $$I\left(n\right)=(1-\beta )I(n-1)$$

Assuming that zero is a desirable harmless value for $I$, then the corresponding value of $\beta$ for the damaging effect to decay to a value significantly similar to zero for a specified relief time can be obtained according to exponential decay using,

9 $$\beta =1-{e}^{-\frac{5\Delta T}{{t}_{r}}}$$

Example, for a relief time of 5 min, ${t}_{r}=300 s$. In this case, with a sampling time of 0.1 s, $\beta =0.0016653$. In the case of CSCM recommendation of 2 min relief time, $\beta = 0.0041580$.

### Pressure–time damaging effect estimator

Instead of performing pressure–time or stress-time integral, data may be used to predict a quantity that can be integrated to indicate the status of a tissue under monitor. This is the purpose of the pressure–time damaging effect estimator, $q(t,P)$ whose output represents an indication of the damaging effect of a given pressure–time combination. Note that the discrete time index, $n$, is not relevant for developing the model of the estimator and is therefore dropped in $q(t,P)$. Clinical experience based pressure–time injury threshold curve exists for humans [23] but clinical data do not exist to quantify an overall damaging effect of a given pressure–time combination. However such clinical data for estimation of a pressure–time overall damaging effect exist for pigs whose skin has certain characteristics similar to humans [32]. Therefore, to demonstrate the method presented here, the pressure–time data from Daniel et al. 1985 was used to model the damaging effect due to a given pressure–time combination assuming that the damaging effect (damage in [32]) is a continuous scale type variable. The sample data, excluding an outlier observation which also did not correspond to tissue damage, were fitted using the generalised linear model of the form,

10 $$q\left(t,P\right)={c}_{0}+{c}_{1}t+{c}_{2}P$$

where the pressure $P kPa$ is applied for a duration $t min$ with a practical consideration that$P>0$. Model selection was verified in MATLAB (version R2020b) using *stepwiseglm* and the fitting was performed using *fitglm*. The model was statistically significant, R^2^ = 0.6476 (adjusted R^2^ = 0.5934), F(2,13) = 11.9435, *p* = 0.0011. The duration and pressure magnitude terms were estimated to be c_1_ = 0.14938/minute (t-stat = 4.8837, *p* = 0.00029865, 95% CI = 0.0833 – 0.2155, SE = 0.030588), c_2_ = 0.0014787/kPa (t-stat = 3.3372, *p* = 0.0053512, 95% CI = 0.0005 – 0.0024, SE = 0.0004431) respectively. The intercept term, c_0_ = 0.55323, did not reach significance at 0.05 level (t-stat = 1.1514, *p* = 0.27029, SE = 0.48047). This result for $q(t,P)$ is used to compute the responses of the monitoring methods later in the results.

### Damaging and non-damaging pressure magnitude

The incremental damaging effect of an applied pressure can be accumulated with a warning raised when a damaging effect threshold is surpassed to suggest pressure relief. However, there is a low pressure limit below which an applied pressure is unlikely to cause clinical damage regardless of the total application time. Likewise an upper limit exists above which an applied pressure can be considered to be instantaneously damaging. For the former it may be unnecessary to accumulate the damaging effect and for the later an immediate alarm may be raised to warn of an instantaneous damaging pressure.

The decision for the lower and upper pressure limits may be made using pressure–time injury threshold curve. A pressure–time injury threshold curve for humans is presented by Reswick et al. [23], but it has been argued that the model may not be entirely accurate especially for high pressures applied at short intervals [24]. The pressure–time injury threshold model presented for albino rat skeletal muscles [25] or engineered muscle tissues [24] may be applicable to human tissues as has been previously suggested [26]. The albino rat skeletal muscle model is given by [25],

11 $$P\ge \frac{K}{1+{e}^{\alpha \left(t-t0\right)}} +C$$

where $K, C, t0$ and $\alpha$ are empirical constants. The lower pressure endurance limit is approximated by the asymptote $P=C$ while the upper limit is approximated by $P=K+C$. Pressure magnitudes within these limits may be accumulated as normal but those outside may be appropriately considered as non-damaging and instantaneously damaging pressure magnitudes. The upper pressure endurance limit, $K+C$ is 32 kPa for albino rats skeletal muscle while the lower limit $C$ is 9 kPa [25]. Similar thresholds values can be stated, perhaps through clinical experience, for human tissues for the purpose of deciding if a given pressure magnitude is instantaneously damaging or non-damaging. A lower threshold value of $C=9 kPa$ may also be reasonably applicable in able-bodied humans. Similar animal data have previously been used in human studies [26]. This result for damaging and non-damaging pressure magnitude is used to compute the responses of the monitoring methods in the results section.

## Results

### Responses of the monitoring approaches

**Fig. 1:**
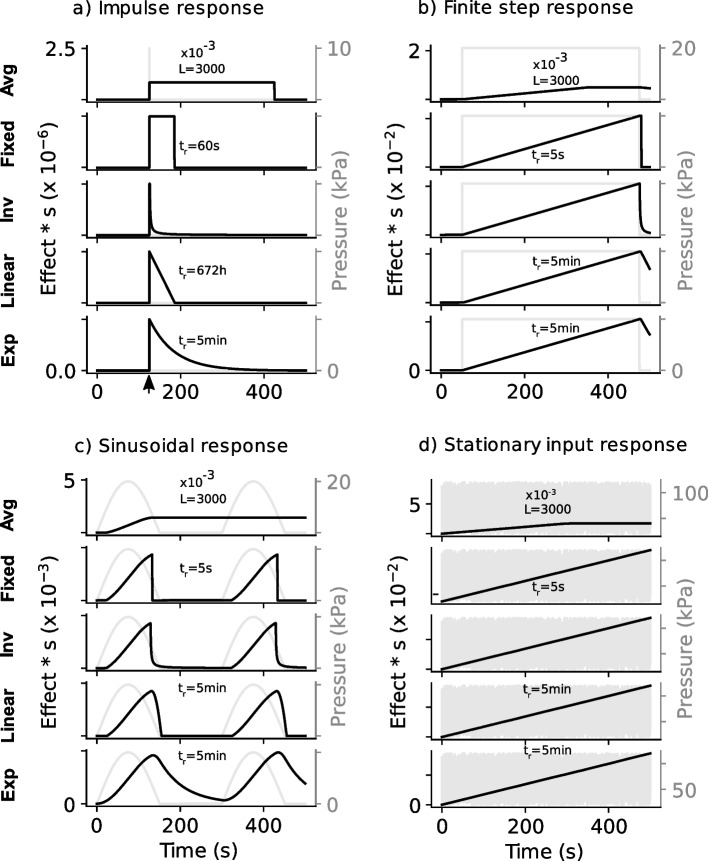
Responses of the monitoring methods to simulated input pressure signals. Parameter settings include sampling time, 0.1 s and relief time (tr), as shown in the figure. Pressure magnitude was set to 10 kPa in **a**), 20 kPa in **b**), 20 kPa amplitude at a frequency of 0.00333 Hz in **c**), and varied between 20 and 120 kPa with a mean of 70 kPa in **d**). In all cases the none-damaging and excessive pressure magnitude were 9 kPa and 32 kPa respectively according the pressure–time injury threshold. The arrow in the last row of **a**) indicate the impulse time. Avg, Averaging filter method; Fixed, Fixed decay with integrator method; Inverse, Inverse time decay with integrator method; Linear, proposed monitoring approach with linear decay; Exp, proposed monitoring approach with exponential decay

An averaging filter [19], inverse [30] and fixed time [15, 26] methods have been used in tissue load monitoring for prevention of PU development. The proposed monitoring approach (integral method with linear or exponential decay approach) presented here were examined together with the previous methods to demonstrate their typical use. Figure 1 shows the responses of the examined methods when simulated pressure load was applied. The code for the simulation is available on GitHub at https://github.com/BethelOsuagwu/pmonitor. Since the relief time has a different implication for the examined methods, it was chosen independently to allow the shape of the response of each examined method to be studied. For the averaging filter method the filter length was set to 3000 samples. For the fixed method the relief time was set to either 60 s or 5 s. For the proposed monitoring approach, the relief times were determined using the described methods with U set as$\frac{\mathrm{min}(\left[q\right],{q}_{m})}{{q}_{m}}$, where $\left[.\right]$ indicates a mean value and ${q}_{m}$ is the output of the damaging effect estimator corresponding to applying the lowest damaging load/pressure threshold for one sample period as shown above. Equations 10 and 11 were used respectively for the damaging effect estimator, and damaging and non-damaging thresholds respectively. Sampling period was set to 0.1 s. Pressure magnitude was set to 10 kPa in Fig. 1a), 20 kPa in b), 20 kPa over a sine wave with a frequency of 0.00333 Hz in c), and varied between 20 and 120 kPa with a mean of 70 kPa in d). The impulse response of the averaging filter (Fig. 1a Avg, row 1), implies that, following a relief, the impact of an applied load on a tissue remains fixed until a time, equal to the relief time (equivalent to filter length) has elapsed after which instantaneous total recovery occurs. For the fixed method (Fig. 1a Fixed) with a relief time ${t}_{r}=60s$ and inverse method (Fig. 1a Inv), an accumulated damaging effect decays as quickly as the load relief occurs. These are however unlikely since the tissue may require some time to gradually recover following the relief as a result of accumulated damaging effect of even a small repeatedly applied load which can facilitate development of a PU [32]. The proposed monitoring approach with linear or exponential decay demonstrated in Fig. 1a (Linear & Exp) may potentially represent a more accurate recovery rate following a pressure relief. The verification of a tissue recovery rate is however not within the scope the current work.

**Fig. 2:**
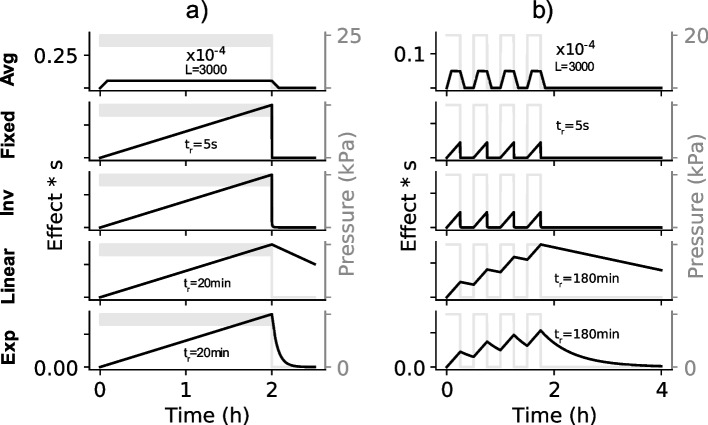
The responses of the monitoring methods to a prolonged simulated loading using recording sampling time of 0.1 s. **a** Responses to a simulated pressure input ranging between 20 – 25 kPa. **b** Responses to a simulated repetitive input with period 30 min and range between 0 – 20 kPa. See Fig. 1 for the description of the annotations

Figure 1b-d demonstrate the accumulation of the damaging effect for the monitoring methods on different input signal patterns. The results are different for the averaging filter which demonstrates the disadvantage of this method which does not use an integrator (Fig. 1b-d Avg). It can be seen from these figures that after a short period the averaging filter stopped accounting for additional impact of an applied stationary load. This means that the averaging method is not adequate for long-term monitoring to indicate the impact of an applied load over a prolonged period. This disadvantage of the averaging method relative to the integrator is demonstrated further for a typical loading period in Fig. 2.

Figure 2a demonstrates the responses of the monitoring methods for a typical monitoring period with a simulated pressure load of about 20–25 kPa which may be applicable during a prolonged wheelchair sitting without regular pressure relief. Figure 2b demonstrates the responses of the monitoring methods for a simulated repetitive loading with a period of 30 min. Repetitive loading may be experienced by wheelchair users during dynamic locomotion [33] or sporting activities [34] and also with active support surfaces that cyclically distribute pressure. In both cases (Fig. 2a-b) the proposed monitoring approach (i.e. Exp and Linear in Fig. 2a-b) accumulated the overall impact of the varying and repetitive loading. The disadvantages of the Fixed and Inverse decay methods can be seem in Fig. 2b, where for a repetitive input, the methods may show a zero overall damaging effect after a prolonged period.

After the input pressure was relieved down to a non-damaging magnitude, the accumulated damaging effect decayed linearly and exponentially in accordance with the relief time respectively for the linear and exponential decay methods (Fig. 2a-b). This shows that the output of the proposed monitoring approach may be used to map the effective impact of the overall loading over the prolonged period taking into account any pressure relief along. On the other hand, such a map may not always be possible using the output of the averaging filter with a length as large as 3000, or the Fixed method with a relief time of 5 s, and the Inverse methods.

### Suggested monitoring scheme

**Fig. 3:**
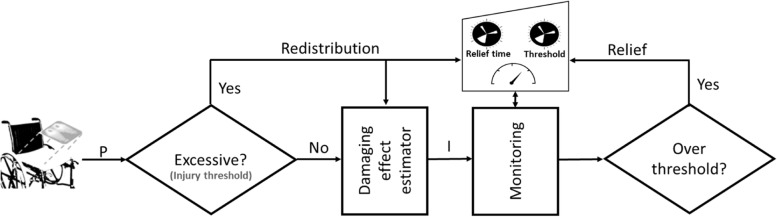
The monitoring scheme for redistribution and relief to prevent ulcer development

A monitoring scheme summarised schematically in Fig. 3 may be developed using the methods proposed here as follows. A monitoring system receives an input interface pressure/internal stress or strain, $P$, at a regular interval and performs a check to determine whether $P$ is considered to be excessively high using an injury threshold model, e.g. the pressure–time injury threshold model. If the check demonstrates that the input is excessive then an immediate alarm is raised to suggest pressure redistribution. Regardless of the damaging status of the input pressure, its impact is estimated using a damaging effect estimator e.g. the pressure–time damaging effect estimator. The overall damaging effect status accounting for the present input and also relieved effect is determined using the proposed monitoring approach. If the accumulated damaging effect surpasses a set threshold, an alarm is raised to suggest pressure relief as shown in Fig. 3.

## Discussion

Real-time pressure monitoring is essential in PU prevention especially in the case of DTIs. A considerable research effort is devoted to determining the magnitude of the distress on a tissue due to a mechanical load. However, there is no established standard method of determining the effective clinical damage or the risk thereof due to the distress. A formalised real-time pressure monitoring approach that tracks the net damaging effect of applied mechanical load over a prolonged period may be utilised for this purpose. Here, an applicable pressure magnitude and duration monitoring approach is proposed, which can be used to accumulate the damaging effect of a continuously or repetitively applied pressure allowing its output to be used to indicate the effective impact of the load over a prolonged period.

### Development of the monitoring approach

The development of the monitoring approach included an integrator as a damaging effect function to accumulate outputs of a damaging effect estimator. An integrator has previously been used with finite element model analysis derived internal compression stress to obtain ‘stress dose’ in human studies [22, 26]. It has been demonstrated in a study using sustained deformation of an engineered muscle tissue construct under an indenter that the percentage of cell death depends on a load magnitude and time of application [12]. The study demonstrated an accumulation of cell death that may justify the choice of an integrator as a damaging effect function, especially in the first 4 h of a high compressive straining of cells under the indenter. A similar result was obtained in an animal experiment where the tissue damage contributions of deformation, ischaemia and reperfusion were considered [8]. The death of cells in Breuls et al. study and tissue damage indicated by MRI T2 time in Loerakker et al. may not equate to a clinically meaningful PU; and other mechanisms in addition to sustained cell deformation which are known to drive PU formation must be accounted for, to determine an exact nature of damage accumulation. But a simple integrator is likely sufficient here in this usage since the aim is to estimate the accumulated impact of the applied load over time.

Unlike in previous studies [17–19] the damaging effect estimator which determined the impact of a given pressure over a given duration, estimated the relationship between pressure, time and tissue damage using a linear model. The use of such a relationship is likely to lead to improved pressure monitoring. Available data in the literature suggest that a linear model may provide a suitable approximation. For instance, a small sample over a 24 h period from a similar study as that of Breuls et. al [12]., using compressive straining of cell constructs, provided a cell damage curve that may be approximated with a linear trend line (R[2] = 0.95, e.g. see Fig. 6 in [11]).

For the relief function, linear and exponential decay methods were explored in addition to other methods available in the literature. The linear and exponential decay methods made it possible to explicitly determine the relief time. It was demonstrated that these methods (a linear and exponential decay methods) are likely better than the current methods such as the average filter method [19], fixed [15, 26] and inverse time methods [30] found in the literature (see [27] for a review of previous methods). However the linear and/or exponential decay method used here may still not accurately explain tissue recovery following pressure relief especially when reperfusion injury which takes place after pressure relief is considered. But considering only deformation injury induced by an applied pressure perpendicular to the skin, the linear and exponential decay, and the relief time used here may better explain tissue recovery than the current methods in the literature. For example, the length of a moving average filter implicitly determined the relief time in Verbunt et al. [19] and Linder-Ganz and colleagues defined a relief time of 1 s [26] e.g. see Fig. 4 in [26]). An older work by Temes and Harder [15] defined a default relief time of 30 s (range: 5 – 60 s). In Portnoy et al. 2011, [30], the relief time related to the time it took for an accumulated stress dose to decay inversely proportional to a time length (in seconds) of pressure relief. These relief time methods may not be adequate for long-term monitoring and likely do not represent how a tissue recovers from a damaging effect of an applied pressure (Figs. 1 and 2). Data from animal models showing changes in tissue damage (indicated using MRI transverse relaxation time, T2) relative to a preloading threshold is shown to *increase* (see Fig. 5 in [35]) before decreasing *gradually* post-loading [35, 36]. This relief pattern, although not a clinical observation, may not be adequately modelled using the current relief methods in the literature.

The resultant monitoring method can be tuned by choosing parameters including non-damaging and excessive pressure magnitudes, a damaging effect threshold and a relief time. Damaging and non-damaging pressure magnitude may be difficult to set given lack of available related data in humans but they may be estimated from available animal data as in the present work. A damaging effect threshold is the maximum allowed accumulated impact of an applied pressure over a duration. It should be defined such that repeatedly exceeding a set value would result in development of PU. It may be necessary to define a damaging effect threshold for specific individuals and tissues. Selecting an appropriate threshold in patients such as those with SCI will required longitudinal data collection to study the distribution of pressure prior to the development of PU. Such data may be analysed for different types of injuries, tissues and anatomies.

The relief time may be chosen based on the characteristics of an individual or condition, tissue and applied pressure magnitude. A low relief time implies that a tissue would quickly recover from an impact of a pressure following pressure relief; while a large relief time implies that a long period is required for recovery. For a highly varying input pressure such as the repetitive input in Fig. 2b, a large relief time may be required to capture an overall impact. An example of a repetitive input is with commercially available active support surfaces which may redistribute pressure at a frequency of 1/600 Hz equivalent to 10 min cycle duration [37]. If pressure is applied for an insufficient time to produce PU initially on a tissue, the tissue may still sustain a level of damage due to the pressure, which makes it susceptible to further damage from even a small additional pressure [38]. Therefore the accumulated damaging effect of such repetitive/multiple pressure loading may eventually lead to PU [32]. For example, patients at risk of developing a PU demonstrated increasingly lower tissue oxygenation with repetitive loading [39] and dynamic loading, as studied with cyclic shear, suggesting an accumulation of the loading impact which may cause increased distress to a tissue [20, 40]. Moreover, cyclic loading may result in more ischaemia reperfusion than continuous loading and therefore may lead to more reperfusion induced tissue damage, as demonstrated in animal models [9, 20, 41], which may accumulate over time. The proposed monitoring approach can be set adequately with a large relief time to accumulate the damaging effect of a cyclic loading.

### Application

The approach presented is suitable for long-term pressure monitoring, especially when a load sensor has a fixed location on the body such as when seated e.g. in a wheelchair, and when wearing a medical device or orthoses. It can be used to programme an alert system or provide a visual feedback which may be implemented using a smartphone device [27]. A healthcare centre may use the method to keep a record of a tissue’s status which is useful for investigation into the cause of a PU. It can be used to indicate when relief is required due to pressure asserted by a medical device including orthoses and also to objectively implement the Consortium for Spinal Cord Medicine (CSCM) guidelines and NPUAP recommendations [1, 42, 43] as well as able-bodied reference behaviour [44] for relief time and frequency (i.e. repositioning frequency) with respect to a particular individual and tissue. For example, the recommendation of CSCM for wheelchair users with SCI include a relief frequency of 15–30 min and a relief time of approx. 2 min. These values can be used to configure the presented monitoring approach by setting an accumulated damaging effect threshold equivalent to 15–30 min application duration and relief time of 2 min. With this, since effective pressure impact and ongoing relief is accounted for, pressure relief will be efficiently requested as required which may be more, or less frequent than usual.

The benefit of the presented monitoring scheme is the ability to separate the indications for redistribution and relief of pressure. This ensures that the presence of an excessive pressure is dealt with immediately e.g. using pressure redistribution systems such as active support surfaces. It enables repositioning or redistribution using an active support surface to be performed only when required to save time and resources [45]. Unlike the raw pressure/stress data which indicate the current mechanical load, the values from the current implementation indicate the effective impact of a load during and after loading. Since redistribution may not replace physical repositioning [37] and merely avoiding high magnitude interface pressure or internal stress may not necessarily equate to pressure relief due to the factor of time, the scheme provides a mechanism for indicating when pressure relief is required. Following pressure relief, some part of the body may continue to experience pressure [46]; the presented method makes it easy to identify such body areas.

Additionally, with this monitoring method the impact of repetitive loading e.g. those experienced between a residual limb and prosthetic socket, during wheelchair dynamic locomotion [33] and sporting activities [34] or use of active support surfaces, are correctly accounted for.

### Limitations and future work

The study, for simplicity, explored the impact of pressure under fixed tissue characteristics and external factors. This meant that the study did not consider temperature, moisture at the seating interface [47], and other factors. Temperature for instance has been demonstrated to affect PU formation [48, 49], which may explain why there is interest to optimize the thermal properties of sitting support surfaces to avoid PU formation [50]. Future study is required to determine factors that account for temperature and other factors in pressure PU formation. For example, the damaging effect function, the damaging effect estimator, and relief function should be developed to consider temperature, tissue characteristics, and other relevant factors.

The damaging effect estimator was based on a *small sample animal data* [32]. More studies with a large sample size are required to produce a reliable model. Perhaps the required data may be acquired using engineered muscle tissues [24] where damaging and non-damaging pressure magnitudes, tissue characteristics, temperature, moisture and other factors may be studied for a prolonged period.

Although justifiable based on available non-clinical data [11], the linear fit used here for the damaging effect estimator may be unreliable. This is because although the related curve in Daniel et al. 1985 was described as hyperbolic [32], analogous to the pressure–time injury threshold curve of Reswick & Rogers [23], the data was fitted here with a linear model. However, given that Reswick & Rogers’ clinical experience-based curve for pressure–time injury threshold [23] has already been disputed at extreme pressures and times [25], it is possible that with extended data in the Daniels et al. experiment, including low/high loads for shorter as well as longer duration, a non-hyperbolic model that could have a linear approximation may be obtained as suggested by non-clinical data [11].

Further investigation is required to identify suitable relief functions and time. The recommendation from Consortium for Spinal Cord Medicine (CSCM) is a 2-min relief time. This is based on the time required, in SCI individuals, for tissue oxygen levels to recover following pressure relief [1, 31]. Therefore this may be a sufficient time to avoid an ischaemia induced damage. However it is not clear if this time is sufficient for interstitial fluid movement to be restored in these group of individuals to avoid any damage associated with the obstruction of the fluid movement. Also it may not be adequate time for a tissue to recover from ischemia reperfusion injury and direct deformation related damage. For example, in an indenter study of rat models using MRI and histological examination, tissue damage indicated by T2-weighted images demonstrated signs of tissue damage until after 90 min following pressure relief [35]. A similar MRI study also indicated that the reversible damage due to ischaemia may take between 90 min (based on changes in perfusion index) to 2 h (based on transverse relaxation time) to reverse [36]. So although the 2-min relief time may be adequate for reperfusion, it is possibly insufficient for a full tissue recovery following pressure relief. If indeed the relief time is not sufficient for a tissue to recover fully or at least significantly from the damages, then the damages may accumulate on the tissue over time despite regular pressure relief. Such an accumulation may eventually lead to development of PU. To address these issues, further investigation, possibly using animal models are required to evaluate relief functions and identify applicable relief time particularly following relief from a load with reversible induced tissue damage.

It is also important to standardise the monitored signal to identify the individual roles of interface pressure and internal stress/strain measurements, and to incorporate monitoring of shear and friction forces.

## Conclusions

Pressure ulcer is a debilitating condition that disproportionately affects people with impaired mobility which facilitates tissue damage through prolonged unrelieved pressure. Real-time pressure monitoring is a crucial part of PU prevention. It guides decisions on the choice of support surfaces and enables continuous monitoring of a tissue with regard to applied load. A tunable continuous pressure monitoring approach is proposed which provides an indication of the effective impact of a load during and after loading. In addition to prolonged time-integral of the impact of the applied load, the approach accounts for ongoing pressure relief using smooth decaying functions with time as a parameter. The approach may be used for further development to formalised pressure monitoring methods aiming to indicate the risk of PU development in real-time.

## Data Availability

The datasets generated and/or analysed during the current study are available in the GitHub repository, https://github.com/BethelOsuagwu/pmonitor.
